# Adaptive Management and the Value of Information: Learning Via Intervention in Epidemiology

**DOI:** 10.1371/journal.pbio.1001970

**Published:** 2014-10-21

**Authors:** Katriona Shea, Michael J. Tildesley, Michael C. Runge, Christopher J. Fonnesbeck, Matthew J. Ferrari

**Affiliations:** 1 Department of Biology and Center for Infectious Disease Dynamics, The Pennsylvania State University, University Park, Pennsylvania, United States of America; 2 School of Veterinary Medicine and Science, University of Nottingham, Leicestershire, United Kingdom; 3 US Geological Survey, Patuxent Wildlife Research Center, Laurel, Maryland, United States of America; 4 Department of Biostatistics, Vanderbilt University School of Medicine, Nashville, Tennessee, United States of America; Princeton University, United States of America

## Abstract

Optimal intervention for disease outbreaks is often impeded by severe scientific uncertainty. Adaptive management (AM), long-used in natural resource management, is a structured decision-making approach to solving dynamic problems that accounts for the value of resolving uncertainty via real-time evaluation of alternative models. We propose an AM approach to design and evaluate intervention strategies in epidemiology, using real-time surveillance to resolve model uncertainty as management proceeds, with foot-and-mouth disease (FMD) culling and measles vaccination as case studies. We use simulations of alternative intervention strategies under competing models to quantify the effect of model uncertainty on decision making, in terms of the value of information, and quantify the benefit of adaptive versus static intervention strategies. Culling decisions during the 2001 UK FMD outbreak were contentious due to uncertainty about the spatial scale of transmission. The expected benefit of resolving this uncertainty prior to a new outbreak on a UK-like landscape would be £45–£60 million relative to the strategy that minimizes livestock losses averaged over alternate transmission models. AM during the outbreak would be expected to recover up to £20.1 million of this expected benefit. AM would also recommend a more conservative initial approach (culling of infected premises and dangerous contact farms) than would a fixed strategy (which would additionally require culling of contiguous premises). For optimal targeting of measles vaccination, based on an outbreak in Malawi in 2010, AM allows better distribution of resources across the affected region; its utility depends on uncertainty about both the at-risk population and logistical capacity. When daily vaccination rates are highly constrained, the optimal initial strategy is to conduct a small, quick campaign; a reduction in expected burden of approximately 10,000 cases could result if campaign targets can be updated on the basis of the true susceptible population. Formal incorporation of a policy to update future management actions in response to information gained in the course of an outbreak can change the optimal initial response and result in significant cost savings. AM provides a framework for using multiple models to facilitate public-health decision making and an objective basis for updating management actions in response to improved scientific understanding.

## Abstract 2

This Research Article explores the benefits of applying Adaptive Management approaches to disease outbreaks, finding that formally integrating science and policy allows one to reduce uncertainty and improve disease management outcomes.

## Author Summary

If the response to a disease outbreak is poorly managed, lives may be lost and money wasted unnecessarily. Lack of knowledge about the disease dynamics, and about the effects of our control strategies on those dynamics, means that it is difficult to do the best job possible managing such epidemiological problems. Here, we present an adaptive management approach that allows researchers to use knowledge gained during an outbreak to update ongoing interventions, thereby translating scientific discovery into improved policy. We explore the implications of adaptive management for foot-and-mouth disease outbreaks in livestock and for measles vaccination strategies in humans. In these two particular cases, planning to update management actions leads to the recommendation of a less aggressive initial approach than if changes in management are not anticipated. We demonstrate expected savings of up to £20 million in terms of lower livestock losses to culling in a foot-and-mouth outbreak based on the dynamics observed in the UK in 2001. Similarly, up to 10,000 cases could have been averted in a measles outbreak like the one observed in Malawi in 2010. Adaptive management allows real-time improvement of our understanding, and hence of management efforts, with potentially significant positive financial and health benefits.

## Introduction

Improvements in public health and disease control may arise not only from novel technologies, but also through novel strategies for optimal selection and application of existing technologies [1]–[4]. Unfortunately, optimal decision making for management of epidemiological systems is often hampered by considerable uncertainty. The sources of uncertainty are myriad, but can be broadly classified into one of two categories [5]–[7]. Epistemic uncertainties are due to a lack of system or process knowledge (biological or ecological); importantly for decision makers, such uncertainties can be reduced through improvement of the state of information. Aleatory uncertainty, which includes environmental variation and other uncontrollable stochastic events, cannot generally be reduced through learning.

The implementation of epidemiological interventions under epistemic uncertainty usually takes place via one of two distinct approaches. Under non-outbreak conditions, the focus is on reducing uncertainty through research; efficacy and risks associated with novel technologies or strategies are typically inferred from extensive clinical trials [8]. While this experimental approach potentially allows for the strongest inference, it is unlikely to be rapid enough to inform dynamic decision making during a crisis. During a novel crisis, such as a disease outbreak or the emergence of a new pathogen, decisions are usually informed through retrospective analyses of prior crises, trials, and interventions [3],[9]–[12]. However, most relevant information about the dynamics of the current crisis comes from observation of the outbreak as it progresses [13]–[15]. Epidemic management practice does not currently incorporate this real-time information into ongoing decision making in any formal, objective way.

Ideally, we would like to learn while we act, rather than only before or after. In this way, we benefit from real-time feedback from the epidemic, including the response to intervention. Adaptive management (AM) is a structured, iterative, decision-making approach for dynamic problems that acknowledges uncertainty and aims to reduce this uncertainty in order to improve outcomes. AM has a robust history in both conservation and wildlife management [16]–[26], which face an analogous challenge to manage in the face of incomplete knowledge of the underlying system and its dynamics. AM determines an optimal state-dependent policy, given a set of management options, a reward (or cost) function, and one or more state dynamics models. In the face of an epidemic, reducing epistemic uncertainty is justified only when it leads to improved management; learning is not valued for its own sake. AM accounts for the future consequences of current actions by weighing the tradeoffs between short-term learning and long-term management gains; thus evaluation of the outcomes of interventions is an essential step.

Using an AM approach has several key advantages over existing approaches. First, science and policy-making are fully integrated rather than being conducted in a sequential manner; such integration prevents loss of information and reduces the subjectivity in decision making. The formalization of the entire process allows decision makers to take full advantage of the considerable literature on decision theory, with its array of tools for rigorous decision making [27]–[31]. This process requires decision makers to explicitly specify objectives and articulate the scientific uncertainties that impede management, thereby providing important insights into the decision problem from the outset. Uncertainty is addressed explicitly, in a synthetic manner, rather than being ignored or addressed in a piecemeal fashion. Thus, instead of making decisions that are contingent on different individual model formulations and assumptions, the AM framework suggests an optimal decision, or set of decisions, that integrates across all models. Finally, the choice of management actions can be updated in response to current events, in a formal and objective way, rather than being decided *a priori* and then only updated on an *ad hoc* basis when the weight of evidence demands a shift in tactics, if at all.

Despite its potential to improve management, there has been no formal application of planned learning with an explicit strategy for updating interventions (i.e., AM) in epidemiological systems (but see [32]–[36]). We here illustrate the potential utility of AM for two epidemiological case studies: management of foot-and-mouth disease (FMD), and vaccination strategies for measles outbreaks. We further use these case studies to illustrate a range of possible applications of AM in public health settings.

## Methods

AM is used to make decisions in the face of uncertainty that would otherwise impede consensus. AM involves a sequence of steps (Table 1), including the statement of an objective (usually encapsulated in a reward [or cost] function), of possible management options, and of any uncertainties that hinder effective decision making (usually formulated as alternative state dynamic models). All possible model and action combinations are then evaluated in terms of their ability to achieve the stated objective. If all models agree about the best management action, despite disagreeing about the underlying uncertainty, then no further analysis is needed, and the decision can be made. However, if there is disagreement among models about the best action to take, it is possible to quantify how much learning about the “correct” model can be expected to improve outcomes. If the value of learning is sufficiently high, then an initial action can be chosen (on the basis of the highest expected benefit [or lowest expected cost] in light of model uncertainty), but AM plans for this action to be changed should information gained during early interventions reduce our uncertainty about the best model.

**Table 1:** Steps in an adaptive management framework.

Steps of AM	Comments
**Set-up phase**	
A. **Specify management objective** for the problem in consultation with stakeholders	Intervention goals may be to minimize economic loss, mortality or number of cases, or the duration of an epidemic
B. **Identify possible management actions**	
C. **Construct alternative models** to encapsulate key uncertainties, as well as what is known/agreed	Failure to incorporate key uncertainties can result in ineffective decisions, and hence, unsatisfied objectives
D. **Develop a monitoring plan**	Decide what, how, and how much to measure
E. **Evaluate expected consequences of interventions** under alternative models	Forward projection of the alternative models for each of the management actions to generate testable predictions about system outcomes
**Implementation phase**	
F. **Decide management action(s)** based on model outcomes with respect to achieving the management objective	Probing actions to accelerate learning are only favored if they improve management outcomes in the long-term
G. **Implement management and monitor outcomes**	
H. **Assessment of empirical observations against model predictions** provides evidence to reduce uncertainty and update weights on alternative models	Inference to reassess model credibility and updating of weights to improve management in light of new information

Initial movement through the set-up phase is followed by rapid iterative learning in the implementation phase (feedback loop from H to F). An outer feedback loop (from H to A, B, or C) can also arise, with occasional reconsideration of steps in the set-up phase, as necessary (for example, if preliminary management efforts motivate alterations or refinements to objectives, if new stakeholders become involved, if new uncertainties become apparent, or if new management options arise). This slow-rapid iterative learning process is called double-loop learning [21]. The set-up phase can be conducted before an anticipated problem arises (e.g., planning for a possible future outbreak, as discussed for FMD in this paper), while the implementation phase can only occur once an outbreak has started.

The value of AM in selecting an intervention can be evaluated using the expected value of perfect information (EVPI), which estimates the value to the decision maker of resolving one or more uncertainties prior to the implementation of specific decisions. EVPI was originally developed in economics [30], and has since been applied in ecological contexts [30],[37],[38] and in the development and evaluation of clinical trials [39]–[41] to identify key sources of uncertainty that limit management success and direct the allocation of research effort to most efficiently improve management outcomes. EVPI reflects a theoretical maximum achievable benefit [42]. Though managers often passively update interventions as new information comes to light, the potential to recover the EVPI is necessarily limited by the lack of a framework for real-time learning. This explicit structured decision-making framework is integral to AM, in which learning is valued insofar as it helps to maximize the proportion of the EVPI attained through informed interventions.

The EVPI calculates the objective value gained by learning before making a decision. It involves a comparison of costs (and/or benefits) assuming perfect information with costs (and/or benefits) assuming the current level of information. Understanding the value of perfect information can meaningfully quantify the value of undertaking an AM program. Formally, EVPI is the difference between the average of optimum values conditional on each model and the optimum of an average of values, where the expectation is taken over the weights associated with the alternative models:
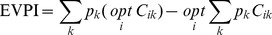
(1)Here, *C_ik_* is the cost associated with action *i* under model *k*, *p_k_* is the weight associated with model *k* (subject to the constraint that 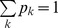), and 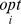 indicates the optimum (in our case, the minimum) over all candidate actions (also see Table 2).

**Table 2:** The costs for the four strategies (IP only [IP], IP+DC [DC], IP+DC+CP [CP], IP+DC+3 km ring culling [RC], when the FMD model is simulated with the dispersal kernels K1, K2, and K3.

Models and Cases	Kernel	Management Actions				
		IP	DC	CP	RC	Best
**Models**	**K1 (thin)**	84.5	**55.1** a	81.7	*259.8* b	55.1
	**K2 (UK)**	*5,129.3*	1,901.3	**1,162.2**	2,537.7	1,162.2
	**K3 (fat)**	284.1	**221.1**	221.1	*381.7*	221.1
**Weighted average costs**						
**Case 1**	UK kernel belief (0.25, 0.5, 0.25)	*2,656.8*	1,019.7	**696.0**	1,429.2	650.1
**Case 2**	Equal weighting (0.33,0.33,0.33)	*1,832.6*	725.8	**540.7**	1,059.7	479.5

The costs shown are in millions of £. Weighted average costs associated with each management strategy for two different distributions of kernel beliefs (case 1, a stronger belief that the 2001 UK kernel will apply to a novel outbreak; case 2, an equal weighting on each model) are also shown.

a Bold numbers highlight the best (lowest cost) outcomes possible.

b Italic numbers are the worst outcomes possible.

We proceed through the AM process for two case studies, using each to illustrate different aspects of value in a range of circumstances. We describe in detail both the set-up (i.e., pre-outbreak) and implementation phases of an AM approach (Table 1) to FMD outbreak response, and quantify the value of a formalized strategy to update management actions as real-time surveillance improves discrimination among models. We illustrate how structural uncertainty (uncertainty about the functional form or parameterization of models) can be characterized by a set of discrete competing models; specifically, we quantify the uncertainty about the spatial scale of FMD transmission. We further quantify the value of a formally adaptive approach to management as the proportion of the EVPI that could be attained and demonstrate that a formal plan to reduce uncertainty can affect the optimal initial intervention. We also explore policy robustness of management recommendations (for example, to scenarios of greater than specified severity, or to very different objectives). We then more briefly sketch the AM approach for measles vaccination planning, using this case study to illustrate the use of the EVPI framework to structure planning when decisions are limited by logistical uncertainties and constraints. This case study allows us to explore a continuum of uncertainty about management capabilities in the field. We further use this case study to explore how the choice of initial action is affected by the time required to monitor management consequences and implement more informed actions.

## Results

### Case Study I: Adaptive Management of Foot-and-Mouth Disease

#### The problem

In 2001 a large outbreak of FMD (*Aphtae epizooticae*), a highly contagious viral disease of livestock, caused major disruption to British agriculture and tourism. There were 2,026 confirmed cases in Great Britain (and four in Northern Ireland); around 7 million livestock (primarily cattle and sheep) were culled [43]. The total epidemic cost was estimated at around £8 billion (US$12.5 billion). The outbreak was characterized by significant, extended, and controversial scientific and political debate about the most appropriate management strategy for the disease; stakeholders included farmers, others in the livestock industry, scientists, and politicians. Passive learning and *ad hoc* changes in management actions occurred during the outbreak, but there was no *a priori* plan to actively include improved understanding of the system dynamics in later decision making. Scientific opinions were encapsulated in three competing models [13],[14],[44], each suggesting preferred strategies to control the epidemic. Here we re-evaluate the problem in a decision-analytical context for alternative versions of one of these models, the Keeling and colleagues [13] model (details of the model are given in Text S1A). We use an AM framework (Table 1) to first outline the set-up phase (Table 1, steps A–E, described in detail below) of the decision process to assess the value of resolving uncertainty about the spatial scale of transmission. We then demonstrate the value of learning in a two-stage AM approach to FMD management, which could be implemented in the event of an actual outbreak.

#### Step A: specify management objective

The primary objective of outbreak management in 2001 was to minimize the number of farms or livestock lost, either through the slaughter of animals on infected premises, or control culling of livestock on farms without reported infection in an effort to control further spread of disease [4],[13],[14]. For the purpose of this work, the objective is to minimize the cost of livestock lost through disease mortality and culling:
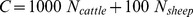
where *C* is the overall cost in pounds sterling, and *N_cattle_* and *N_sheep_* represent the number of cattle and sheep lost to the disease or culled as part of the control strategy. These costs are based upon estimates of market prices of cattle and sheep in the UK from 2001. However, the choice of objective function is critically important: we also consider alternative objectives, below and in Text S1E. As different objectives can significantly change management recommendations, specification of the fundamental objective of management is a nontrivial process, ideally involving input from all stakeholders (Table 1).

#### Step B: identify possible management actions

**Figure 1:**
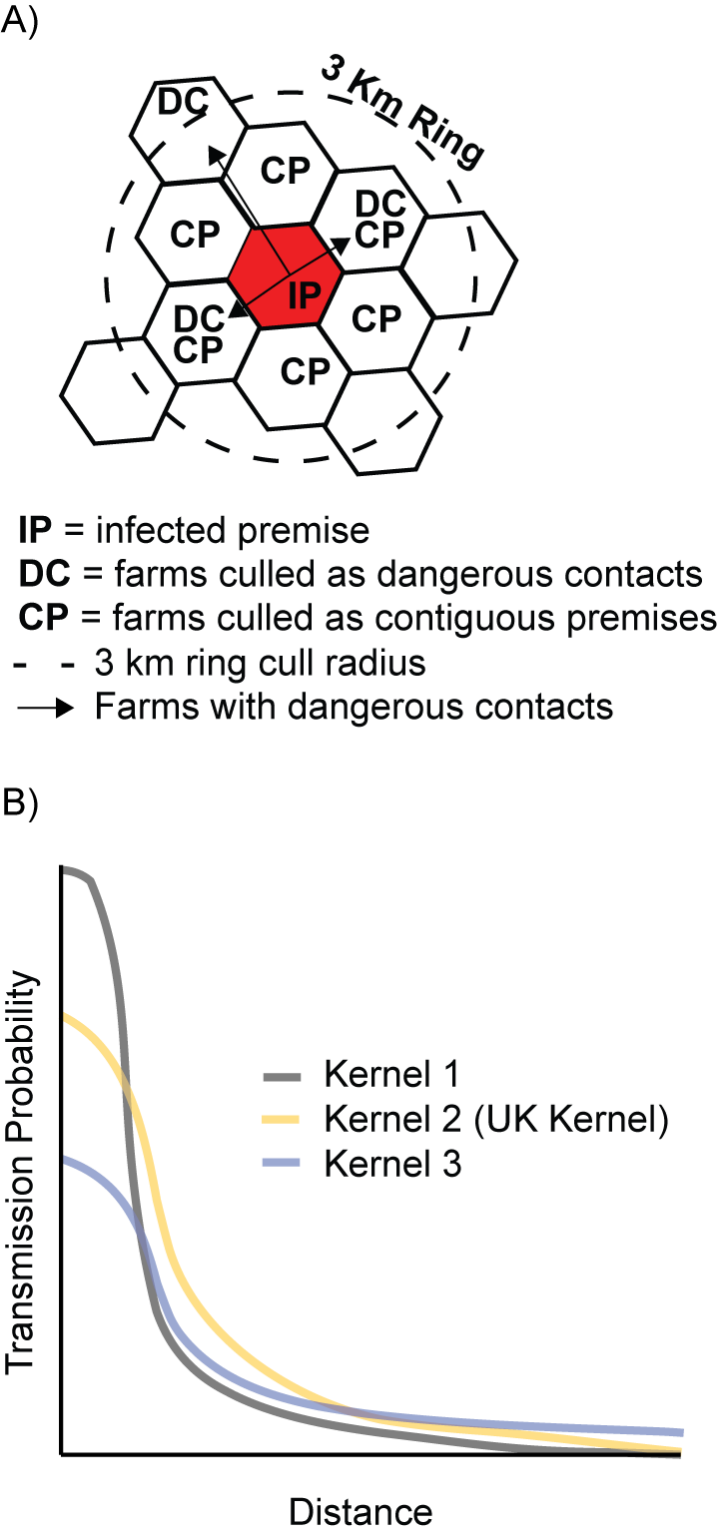
Candidate management actions and alternative dispersal models for the FMD case study. (A) Schematic representing the four possible nested management actions (IP only [IP], IP+DC [DC], IP+DC+CP [CP], IP+DC+3 km ring culling [RC]) for FMD management (corresponding to Table 1, step B). (B) Three alternative dispersal kernel models for FMD in the UK, representing a continuum of possible dispersal kernels (corresponding to Table 1, step C).

We consider four nested management actions (Figure 1A): (1) culling of livestock on infected premises (farms with confirmed cases of disease) only (IP); (2) pre-emptive culling of dangerous contacts (defined either as premises in which animals had been in direct contact with infected livestock or as premises that had been exposed to infection in any other way) as well as infected premises culling (DC); (3) culling of livestock on infected premises, dangerous contacts, and contiguous premises (farms sharing a border with an IP) (CP); (4) ring culling in a 3 km radius of infected premises in addition to infected premises and dangerous contact culling (RC).

#### Step C: construct alternative models to encapsulate uncertainty

The FMD model has 188,496 farms (using data from the June 2000 agricultural census), with an infection process based on demographic characteristics of farms and the distance between farms (Text S1A). We encapsulate the uncertainty in the risk of transmission between farms using three dispersal kernel models, ordered by increasing mean dispersal distance (Figure 1B): a thin, steep kernel (K1); the current estimate from past experience in the UK (K2); and a fat, shallow kernel (K3); we assume the same total force of infection for each kernel. A continuum of model parameterizations that span the possible range could easily be explored, but we focus on these three representative models for clarity. The UK kernel (K2) was estimated using contact tracing from the 2001 epidemic after the introduction of movement restrictions [13]. In the event of future outbreaks of FMD in the UK or elsewhere, the shape of this distance-dependent transmission kernel will be a key source of epistemic uncertainty.

#### Step D: develop a monitoring plan

To rapidly discriminate among competing models, we require information on the key inputs for the cost function (the numbers of cattle and sheep culled), as well as on the location and detection date of infected farms, and contact tracing.

#### Step E: evaluate expected consequences under alternative models using the expected value of perfect information

We assess the four management options against the three alternative kernels in an EVPI analysis of the FMD model [13], using two different kernel weightings (Table 2): (case 1) higher weight on K2, favoring the belief that a novel outbreak in the UK would follow the 2001 dynamics, and (case 2) equal weight on all kernels, reflecting the uncertainty associated with an outbreak under alternative movement restrictions from those imposed in 2001, or elsewhere (e.g., US).

#### Fixed strategies and the expected value of perfect information

Conditional on kernels K1 or K3, a DC strategy over the full epidemic minimizes the expected costs relative to other fixed strategies (Table 2). Under kernel K1, the narrow dispersal kernel contains the epidemic in a small geographic region, while under K3 geographic spread is significant, but the small height of the kernel results in relatively few cases in high density regions. The UK (K2) kernel's combination of significant local and long distance spread results in higher costs regardless of the chosen intervention. Conditional on the UK kernel, the best fixed strategy is CP culling (Table 2). Across the three models, for case 1 and case 2, the CP strategy gives the lowest model-weighted projected cost (£696.0 million and £540.7 million for the two cases, respectively) (Table 2; see also Text S1B). Thus, while the DC strategy is better for two of the three models, the higher costs associated with K2 mean that the CP strategy would result in the lowest expected cost in light of model uncertainty.

Despite IP culling being the least expensive strategy to implement of the four considered here, it is insufficient to curtail FMD spread, even with the narrowest dispersal model assumed, so overall incurs higher costs than DC under all models (hence, IP is “dominated” by DC). RC is also never the best solution, and is the worst strategy for K1 and K3.

**Figure 2:**
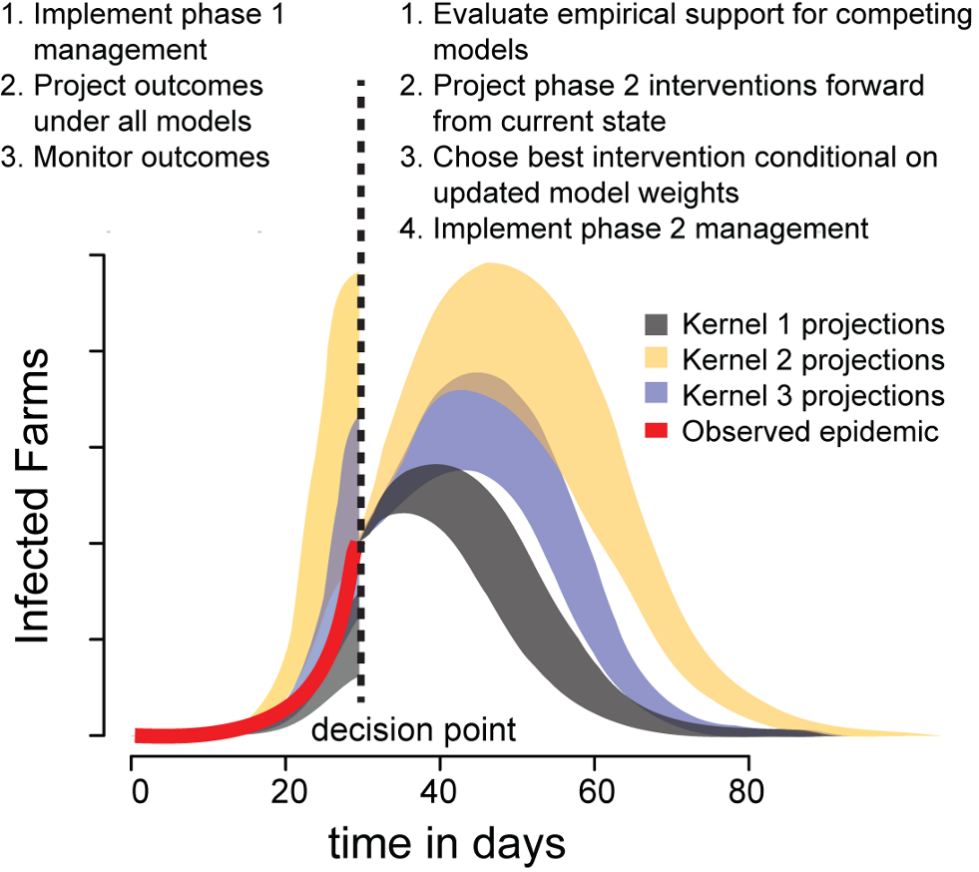
Schematic of the two phases of the AM process (Table 1). Left of the dashed line indicates model projections under the phase 1 management action prior to the first opportunity to update management—colored shading indicates projections of competing models. The red line indicates the observed time series of the epidemic up to the decision point. Right of the dashed line indicates new projections from each model, conditional on the observed epidemic up to the update, from which to assess the alternative management actions.

**Figure 3:**
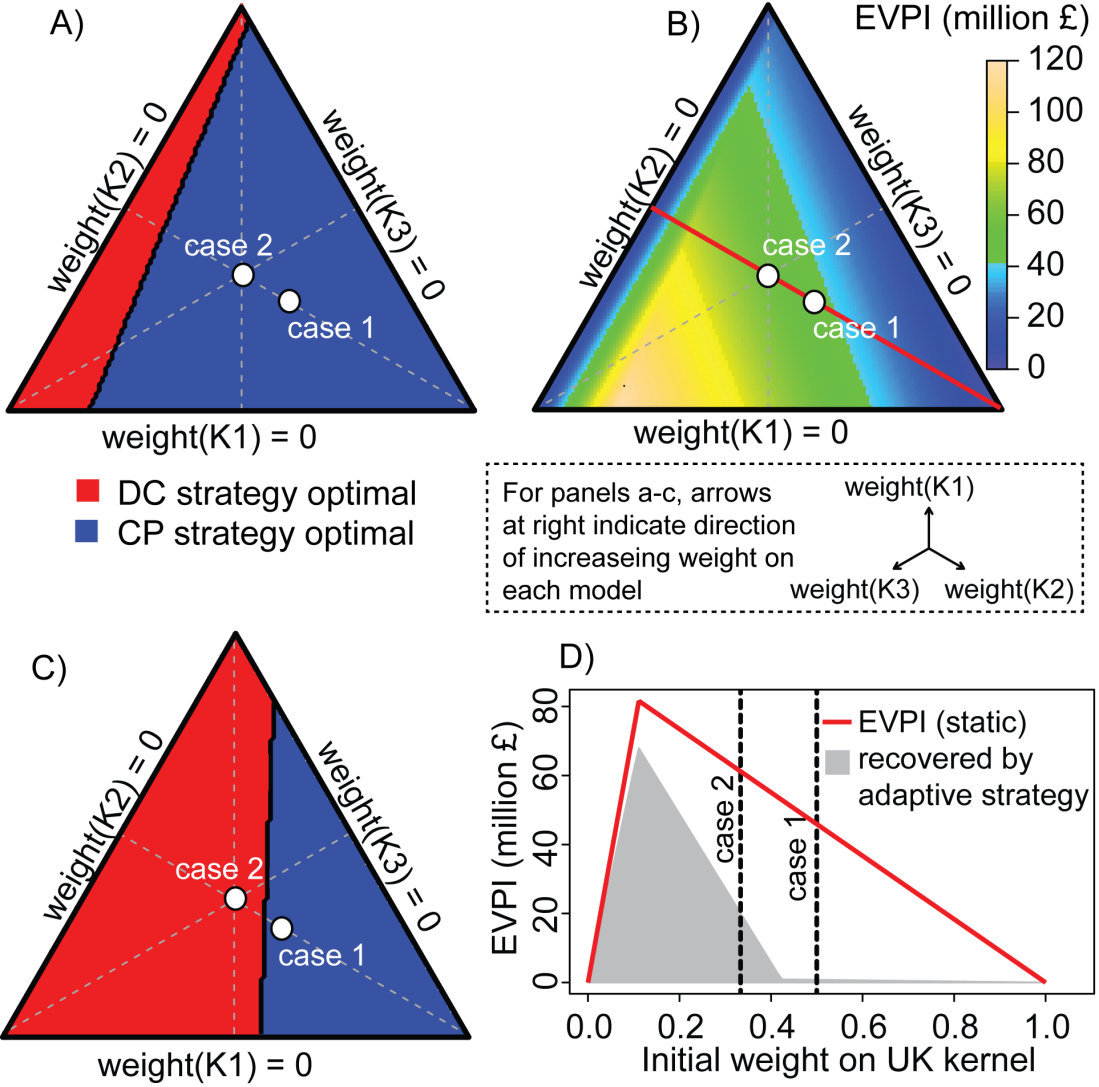
Analysis of the foot-and-mouth case study. (A) The optimal static culling intervention (red, DC; blue, CP) for FMD as a function of the initial weights on the three kernel models. The axes indicate weights on each of the three kernel models and internal points correspond to combinations of weights (summing to 1) on each model; the inset indicates the direction of increasing weight on each model (e.g., the transition from the left axis to the bottom right apex indicates increasing weight on K2 with equal weight on K1 and K3); dots indicate the weight combinations corresponding to case 1 and case 2. (B) The EVPI as a function of the initial weights on the three kernel models; axes and dots are as in (A). (C) The optimal first-stage culling intervention (red, DC; blue, CP) for the two-stage adaptive strategy as a function of the initial weights on the three kernel models; axes and dots are as in (A). (D) The EVPI (red line) as a function of the weight on the UK kernel, assuming equal weights on the other two models. The grey shading indicates the amount of the EVPI that is expected to be recovered under an adaptive strategy.

If we could resolve uncertainty prior to committing to an action, we would choose the best action under the true model. Conditional on the *a priori* model weights, the expected costs of the best strategy are £650.1 million and £479.5 million for case 1 and 2, respectively (“Best” column in Table 2). For a novel outbreak for which prior belief in the transmission dynamics is weighted in favor of behavior consistent with the 2001 UK outbreak (case 1), the EVPI is £45.9 million (£696.0 million–£650.1 million). Thus, the expected cost of a future outbreak could be reduced by £45.9 million (6.6%), relative to the cost incurred by choosing the action recommended by the *a priori* weights, if the uncertainty could be fully resolved before a management action is decided. For a novel outbreak for which prior belief in the transmission dynamics does not support one kernel model more than another (case 2), EVPI is £61.2 million ( = 540.7 million–479.5 million), which is an expected 11.3% cost reduction. In practice, uncertainty in these alternative kernels is unlikely to be fully resolved prior to a novel outbreak (Figure 2). Thus, the best fixed strategy will be conditional on the remaining weights (Figure 3A), and the resultant EVPI (Figure 3B) quantifies the economic incentive for implementing an AM plan.

#### Realizing the expected value of perfect information: a two-stage adaptive management approach

Detection of a new outbreak would trigger the iterative implementation phase (Table 1, steps F–H). For heuristic and practical purposes, we here consider a two-stage management strategy (Figure 2), allowing for control tactics to be updated at a single point one month into a new epidemic, assuming that monitoring during the first month identifies the true dispersal kernel model. We also assume that there is no cost to changing management actions. With one decision point, the four culling options give rise to 16 possible combined strategies, each composed of one (though possibly identical) option in each stage (Text S1C).

For case 1 (2001 UK kernel biased) the best stage 1 tactic in an adaptive strategy is CP, and the expected costs are £695.3 million. Given that the best fixed strategy is CP (Figure 3A) and the best adaptive strategy is CP in the first stage (Figure 3C) the potential gain of an adaptive approach is only 1.7% of the EVPI (£0.78 million). The bulk of the £45.9 million EVPI can only be regained if uncertainty is resolved earlier in the epidemic.

If *a priori* weights are equal for all models (case 2), the best fixed strategy is CP (Figure 3A) but the best adaptive strategy is DC culling in the first stage (Figure 3C) and the potential gain of an adaptive approach is £20.1 million (32.85% of the EVPI). Thus, for a novel outbreak, which may not necessarily progress similarly to the 2001 UK outbreak, the potential gains from an adaptive approach are significant.

Given uncertainty in the spatial scale of transmission, the optimal adaptive strategy and the potential cost reductions arising from an adaptive approach depend on the initial weights placed on the different transmission kernels (Figure 3C and 3D). Increased initial weight on K2, with its higher associated costs, means that the model-weighted expectation of the CP tactic would be favored. EVPI peaks at low *a priori* weight on the UK (K2) kernel (assuming the remaining weight is evenly split between K1 and K3) (Figure 3D); in general, the more certain we are about any individual model, the less the expected value of potential learning. The amount of EVPI that can be recovered by an adaptive strategy (Figure 3D, shaded region) drops quickly, from >80% when the weight on the UK kernel is less than 0.13 to <2% if the weight on the UK kerned is >0.41 (Figure 3D). Such analyses can be used to determine whether an adaptive approach is likely to be justified relative to *a priori* uncertainty in the outbreak scenarios and the costs associated with monitoring, evaluation and adaptation.

**Figure 4:**
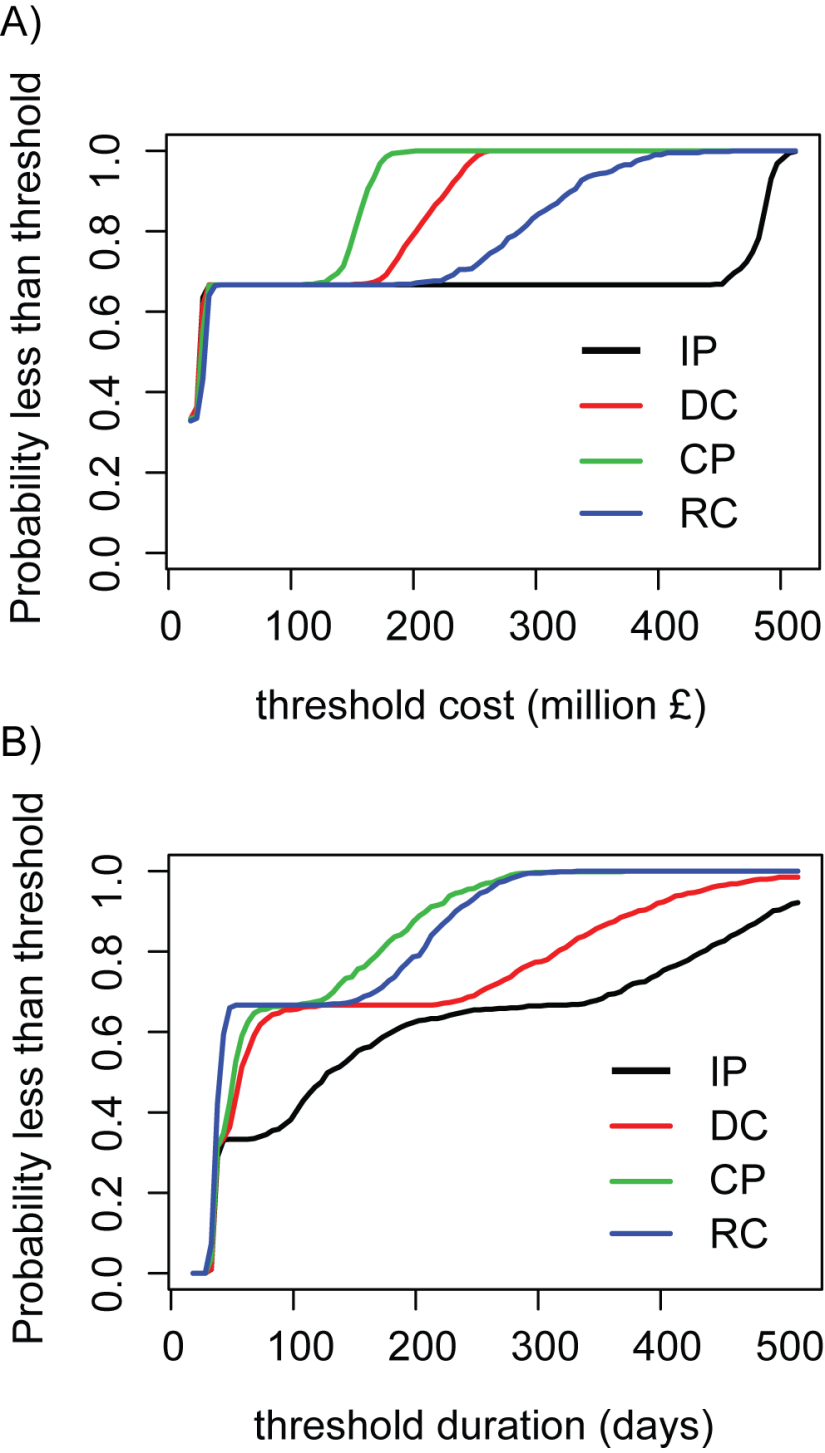
The probability of FMD epidemic outcomes below a stated threshold for the four nested alternative management tactics. (A) Outcomes for the management objective to minimize total epidemic cost due to livestock loss assuming equal weights on the three kernel models. (B) Outcomes for the management objective to minimize the duration of management activities, again assuming equal weights on the three kernel models. The x-axis indicates the cost (in millions of pounds) or duration (days) threshold that managers would like to stay below. The y-axis indicates the probability, averaged across all three kernel models, of outcomes below the threshold for each management tactic (solid lines).

The expected value formulation implies that the goal of management is to maximize the average benefit (or minimize average costs). However, this objective does not explicitly account for variation in outbreak outcomes; the distribution of potential outcomes may be strongly skewed for epidemics, and alternative expressions of the objective might aim to minimize the risk of catastrophic events such as particularly extreme outcomes. It is straightforward to evaluate management alternatives relative to a manager's risk tolerance (i.e., risk prone or risk averse), by stating an objective that maximizes the probability that the outcome is less than some threshold (Figure 4A). Management actions can then be assessed with respect to their “robustness” to these different statements of cost objective (to minimize average costs versus minimization of chance of extreme costs). It is also possible to conduct an examination of very different alternative objectives. For example, minimization of FMD outbreak duration may be more important than minimization of local costs for countries involved in significant international trade (Figure 4B). For both objectives, CP culling either maximizes, or results in the same, probability of remaining below the threshold cost or duration for the majority of possible thresholds, but if threshold duration is low, then the more aggressive RC alternative is most likely to stay below the threshold (Figure 4B), and the relative ranking of the suboptimal RC and DC actions switches for these two objectives (Figure 4A versus 4B). We further describe analyses of alternative objectives in Text S1E.

### Case Study II: Adaptive Management for Measles Outbreaks

#### The problem

Measles is the leading cause of vaccine-preventable childhood mortality worldwide [45]. In 2010, following years of successful control, an outbreak of measles spread throughout Malawi resulting in >135,000 new cases [46]. The Ministry of Health initially implemented a vaccination campaign targeting children aged 6 months to 5 years. After early surveillance indicated that the outbreak was affecting a much broader age range than anticipated (cases up to 25 years of age) [46] the Ministry of Health collaborated with Médecins Sans Frontières to expand vaccination campaigns in eight districts to target children aged from 6 months to 15 years. The age-distribution of the susceptible population is a key uncertainty limiting the design of optimal interventions. For non-selective campaigns, a larger age-range should increase the chances of reaching non-immune individuals; however, it should also result in more resources spent on vaccinating those already immune and necessarily increases the time to implement a campaign. The delay in the completion of vaccination could limit the potential impact of a campaign and the ability to rapidly respond elsewhere if an outbreak spreads [11].

#### Measles set-up phase

Here we consider a simplified example of deciding the age-target for a measles vaccination campaign with the objective (Table 1A) of minimizing the total number of cases over the full duration of the outbreak. The three possible management actions (Table 1B) target all children from 6 months to 5 years, 6 months to 10 years, or 6 months to 15 years of age.

**Figure 5:**
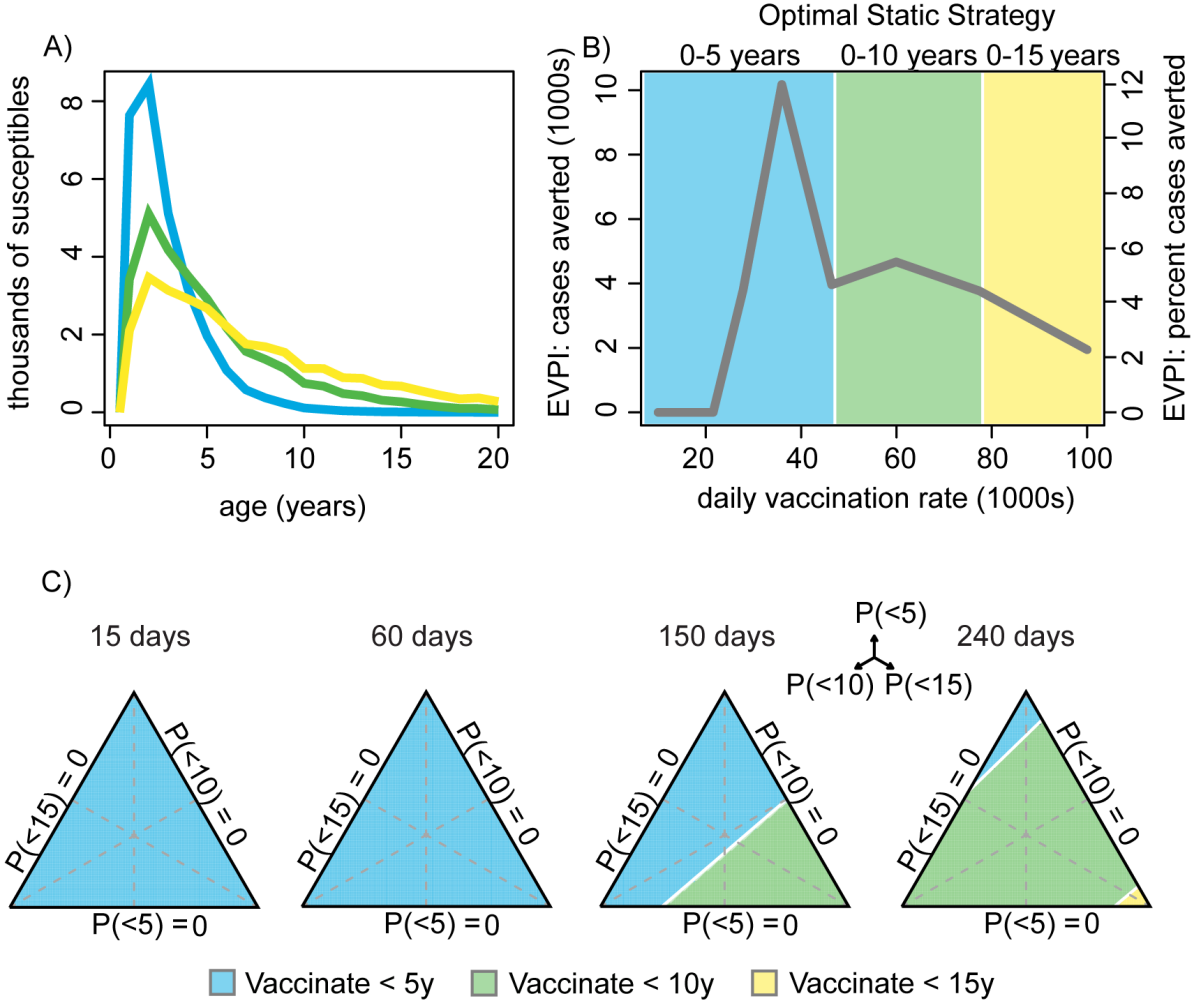
Analysis of the measles vaccination case study. (A) The number of susceptible individuals in each age class for the three alternative age distribution models for measles. (B) The EVPI as a function of the daily vaccination rate, colors indicate the optimal intervention (blue, vaccinate 0–5 years; green, vaccinate 0–10 years; yellow, vaccinate 0–15 years) for each vaccination rate. (C) The optimal vaccination target age, as a function of the weight on each of the three models of the susceptible population (axes on ternary plots) and the time, in days, at which the vaccination target can be changed to the optimal target conditional on the true susceptible age distribution (colors are as in panel B).

We assume a deterministic SEIR-type, age-structured epidemic model (see Text S1D for model details) and calculate the number of measles cases averted (i.e., cases assuming no campaign minus cases with a campaign) by a reactive vaccination campaign that aims for 90% coverage in the target age classes. Unlike the FMD example above, here we consider that the logistical capacity to implement a vaccination campaign is unknown and conduct an *a priori* evaluation of the potential benefits of an adaptive approach. The duration of a campaign is determined as the size of the target population divided by the daily vaccination rate. It is unlikely that the vaccine distribution rate, which depends on both clinic capacity and visitation rate, will be known *a priori*. We consider uncertainty about vaccination rates over a continuous range from 10,000 to 100,000 doses per day. We represent uncertainty in the susceptible age distribution, analogous to the setting in Malawi in 2010, as three alternate models (Table 1C) of the susceptible age distribution (Figure 5; see the Text S1D for details): an exponential age distribution with 90% of susceptibles less than 5 years, 10 years, or 15 years (Figure 5A). We assume that all three age distributions have equal *a priori* weight. In an adaptive approach, the monitoring plan (Table 1D) would facilitate resolution of this uncertainty through case-based surveillance or targeted serological surveys. We next evaluate the expected consequence of interventions (Table 1E); detection of a real outbreak would then trigger the iterative implementation phase (Table 1F–H).

To explore what might happen during a real outbreak, we examine two different scenarios pertaining to the logistical capacity to implement a vaccination campaign. We first assume a campaign conducted on day 75 of the outbreak and calculate the optimal fixed age-target and the expected value of resolving uncertainty about the susceptible age distribution (EVPI), dependent on the daily vaccination rate. We then assume a fixed daily vaccination rate of 30,000 doses per day (approximately that achieved in Malawi in 2010) and calculate the optimal initial strategy assuming that age targets are updated following a delay of T days (T = 15, 30, 60, 180, 250).

In the face of uncertainty about the susceptible age distribution for measles, the optimal initial response depends both on the logistical capacity (i.e., the daily vaccination rate) and the time required to assess and implement an updated age target. When vaccination rates are low the best fixed strategy is to vaccinate only <5 years, and the potential to improve outcomes by learning (EVPI) is low because broader campaigns (up to 10 years or 15 years) would be prohibitively slow even if there were many older susceptible individuals (Figure 5B). The expected benefit of resolving uncertainty about the age distribution of susceptibles (EVPI) is highest (∼10,000 fewer cases, or a 12% case reduction relative to the best naïve strategy which results in ∼84,000 cases) at intermediate daily vaccination rates (∼35,000 doses per day), since switching to a broader age target, if prescribed, would not incur prohibitive delays in campaign completion (Figure 5B). At high daily vaccination rates the duration of the campaign is less constrained by logistical capacity; thus wider age targets are recommended and the potential to improve upon the fixed strategy by learning declines (Figure 5B). Thus, while an adaptive approach always allows actions to be tailored to the current setting, the benefit of an adaptive strategy is constrained by the logistical capacity to implement the recommended changes.

When the daily vaccination rate is fixed, the time required to assess and implement more informed actions affects the choice of initial action. If the campaign age target can be rapidly updated (within 90 days) the best initial action is to target children <5 years, regardless of the initial weight on each model (Figure 5C). The ability to update actions limits the potential costs of the smaller initial age target being incorrect. The EVPI associated with an adaptive strategy is small when updates are implemented within 15–60 days (6%–10% reduction in cases) because the lack of information only impacts decisions for a short period. However, an adaptive strategy updated between days 15–60 can realize 90%–100% of the EVPI and a reduction of burden by 12,000 to 19,000 cases relative to a static strategy that applies a single age target throughout. As the time required to update the initial action increases, then the more conservative strategy of vaccinating children <10 years dominates for all possible model weightings (Figure 5C).

## Discussion

The disparate predictions of competing models are a barrier to the development of policy under traditional (non-adaptive) management approaches [47],[48]. Rather than conditioning on a single “best” model, AM incorporates and systematically seeks to reduce the scientific uncertainty that impedes success, by integrating over models that encapsulate all of the articulated uncertainties to produce an inclusive decision set. Our simultaneous consideration of three alternative parameterizations of the dispersal kernel of the Keeling and colleagues [13] model and all possible interventions illustrates the expected value of resolving uncertainty about the dispersal kernels in an FMD outbreak, possibly saving millions of pounds in lost livestock. Passive learning and *ad hoc* adaptation did occur during the 2001 outbreak (the initial DC strategy was altered to CP within about a month); thus there would be no additional logistical burden to an AM approach. Our results show that an AM approach could be employed to realize a good portion (32.85% for case 2) of the EVPI, and provides an objective justification for an initially less-severe culling regime by minimizing expected costs over the full epidemic, given the option to change management actions in response to the observed progression of the outbreak. As seen in the UK in 2001, FMD outbreaks can potentially cause significant economic and environmental damage, and there is substantial concern about the likelihood and potential impact of future FMD disease outbreaks, both in the UK and the USA. Using an AM approach could significantly reduce the burden of such an outbreak.

The AM approach to the measles outbreak response case study illustrates how management decisions can be framed in the context of both discrete and continuous uncertainty, here with regard to the population at risk and logistical capacity. In particular, our simulations show that the cost of uncertainty about the at-risk population is critically dependent on the logistical capacity to implement the optimal vaccination target. When daily vaccination rate is highly constrained, the optimal strategy is to conduct the smallest, and thus fastest, campaign; however, it is in this regime where the value of information is greatest—potentially reducing case burden by 12% (∼10,000 additional cases averted) if campaign targets can be updated based on the true susceptible population. Further, we illustrate the inherent trade-off between the benefit of updating vaccination targets conditional on assessment of the true susceptible population and the time required to make such an assessment. If vaccination targets can be rapidly adjusted to the outbreak setting at hand, then the optimal strategy is to implement the smallest, fastest initial age target—with the potential to realize nearly 100% of EVPI (which corresponds to 40%–60% fewer cases relative to the best static age target) if updated within 30 days. However, if the initial target cannot be updated (or only updated after a very long period of surveillance), then the optimal recommendation is to choose a broader age target, which averages risk over the alternative distributions of susceptible individuals.

The goal of AM is not to replace decision makers or to automate decision making. Modeling plays an important role in developing a mechanistic understanding of the processes that give rise to observed dynamics and that mediate the costs and benefits of management actions. With an improved mechanistic understanding of a system, inherent trade-offs in decision making can be understood and management can be optimized, in the classical sense of optimal control, relative to a given model. AM plays a role in the common situation where a mechanistic understanding cannot be resolved *a priori*; thus managers must choose among the potentially disparate recommendations of alternative models or parameterizations. In this setting, EVPI is a measure of the degree of consistency between model predictions with respect to management actions. Interpreting EVPI in the context of the full decision-space highlights the dependence of the recommended actions on the underlying models and focuses attention on the differences among models in terms of their recommendations (the management action to best achieve the objective) rather than in terms of the projections of the system states.

AM improves management outcomes in three ways. First, the outcome of management is quantified in terms of an objective function that can be expressed in terms of both desired biological and economic outcomes. Second, the potential benefits of future improvements to management are balanced against the short-term costs of learning [37] and the capacity to enact updated interventions. Third, the expected benefit of initial interventions is calculated in light of the ability to implement future changes; thus there is no *a priori* presumption of a “best” intervention, and management may change through time. Managers often “adapt” their actions on an *ad hoc* basis, but AM formalizes this process by assessing all models and management options simultaneously.

Our case studies demonstrate that AM has the potential to improve management outcomes for a variety of epidemiological systems. The FMD case study showcases the value of AM for improving management interventions as information accrues, rather than relying only on prior knowledge, and anticipates the value of information in choosing early intervention strategies, here via an EVPI analysis. In this example, a more moderate initial culling intervention is optimal for a broader range of parameter uncertainty when the ability to change is included in the analysis. The use of AM in the event of future FMD outbreaks, in the UK, the USA, or elsewhere, would likely also realize significant socio-economic savings. In the measles example, we illustrate that while the expected cost of an adaptive strategy is always less than that of a single fixed strategy, optimal vaccine targets and the additional benefit of an adaptive approach depend both on uncertainty about the age-distribution of the at-risk population and on the logistical constraints of implementing improved interventions. These examples, taken together, illustrate that AM explicitly values enhanced scientific understanding in terms of its capacity to improve management outcomes through selection of appropriate interventions.

AM is flexible and can easily accommodate alternative objectives, additional management options, other models, and multiple sources of uncertainty. For example, other costs, such as damage to the agricultural or tourism industries, could also be included in the FMD objective cost function. Similarly, entirely different objectives, for example minimization of epidemic duration in FMD, to reduce the time taken to return to disease-free status for trade purposes [49] are straightforward to consider (Figure 4; Text S1E). If new management options or models arise, they effectively trigger a return to the set-up phase of AM. For example, vaccination (with its own inherent uncertainties about how, and how well, the vaccine performs) was not implemented during the 2001 FMD epidemic, but is now part of the UK's contingency plan in the event of future outbreaks [4]. Similarly, in future outbreaks under markedly different situations (e.g., in the event of an outbreak in the USA) transmission uncertainty would be even more extreme, and would likely require the assessment of additional kernels (e.g., farm-to-farm contact networks), models, or management strategies [49]. Many management situations also have multidimensional uncertainties. For example, in our analysis of measles we independently examined daily vaccination rate and the rate at which age targets are updated. It is straightforward to weigh the relative value of reducing uncertainty in each of these different unknowns [42],[50]. AM can frame all of these novel aspects. Relative to the analyses presented here, these additional complexities can be readily incorporated by modifying the fundamental objective, or by expanding the value of information analyses (Table 2; Text S1B, S1C, S1E) to include the additional model and intervention combinations (and associated model weights).

Applications of AM are not limited to disease outbreaks. AM also has the potential to improve other disease management outcomes, such as routine and supplemental vaccination strategies, infectious disease surveillance, and clinical trials. AM can improve management outcomes in situations where management actions are taken repeatedly in time or space, system dynamics are influenced by management actions or by changing environmental conditions, and there is uncertainty (or disagreement) about the expected impacts of management. The potential for improvement may be limited by monitoring capacity or by the logistical or political capacity to enact changes. Nevertheless, even if a static intervention is optimal or the value of information is low, the AM approach provides a framework for incorporating predictive modeling into decision making that embraces scientific uncertainty. Thus, AM may yield significant rewards in terms of money or lives saved.

## Supporting Information

Figure S1 **The probability of epidemic outcomes below a stated threshold for four alternative management tactics.** Left panels give outcomes for the management objective to minimize total epidemic cost due to livestock loss, the right panels give outcomes for the management objective to minimize the duration of management activities. The x-axis indicates the cost (in millions of £) or duration (days) threshold that managers would like to stay below. The y-axis indicates the probability, averaged across all three kernel models, of outcomes below the threshold for each management tactic (solid lines). Panels from top to bottom indicate increasing weight on the 2001 UK kernel, with equal remaining weight on kernels 1 and 3.
(TIF)

Figure S2 **Ternary plots of the optimal static strategies assuming different utility functions.** The top row indicates the optimal static strategy for the objective of minimizing total outbreak cost due to livestock loss. The bottom row indicates the optimal static strategy for the objective of minimizing outbreak duration. Each ternary figure indicates the optimal static culling alternative (colors) for different weightings on the three kernel models (see Figure 3 in the main text for description of ternary plots). Panels from right to left indicate utility functions (insets) that are increasingly risk-seeking.
(TIF)

Figure S3 **Ternary plots of the optimal static strategies assuming different utility functions.** The top row indicates the optimal static strategy for the objective of minimizing total outbreak cost due to livestock loss. The bottom row indicates the optimal static strategy for the objective of minimizing outbreak duration. Each ternary figure indicates the optimal static culling alternative (colors) for different weightings on the three kernel models (see Figure 3 in the main text for description of ternary plots). Panels from left to right indicate utility functions (insets) that are increasingly risk-averse.
(TIF)

Table S1 **Cost projections (in millions of £) for each two-stage intervention strategy for each kernel model.**
(DOCX)

Table S2 **Expected cost projections (in millions of £) of each first-stage intervention, conditional on the assumption that model uncertainty is resolved after 1 month and the second-stage action is taken as that intervention that minimizes costs under the true model.**
(DOCX)

Table S3 **Parameterization of the age distribution of susceptibles for three age distribution models.**
(DOCX)

Text S1 **Additional methods and model descriptions.** (A) Description of the Warwick FMD model. (B) Interpretation of the EVPI table. (C) Expected value of an adaptive strategy. (D) Description of the measles outbreak model. (E) Alternate objective formulations to reflect risk tolerance for FMD.
(DOCX)

## Abbreviations

AM: adaptive management
CP: contiguous premises culling
DC: dangerous contacts culling
EVPI: expected value of perfect information
FMD: foot-and-mouth disease
IP: infected premises culling
RC: ring culling
