# Supplementary figures and images for: Adaptive Management and the Value of Information: Learning Via Intervention in Epidemiology
Source: PLoS Biol. 2014 Oct 21;12(10):e1001970. doi: 10.1371/journal.pbio.1001970 (PMC4204804; doi:10.1371/journal.pbio.1001970)

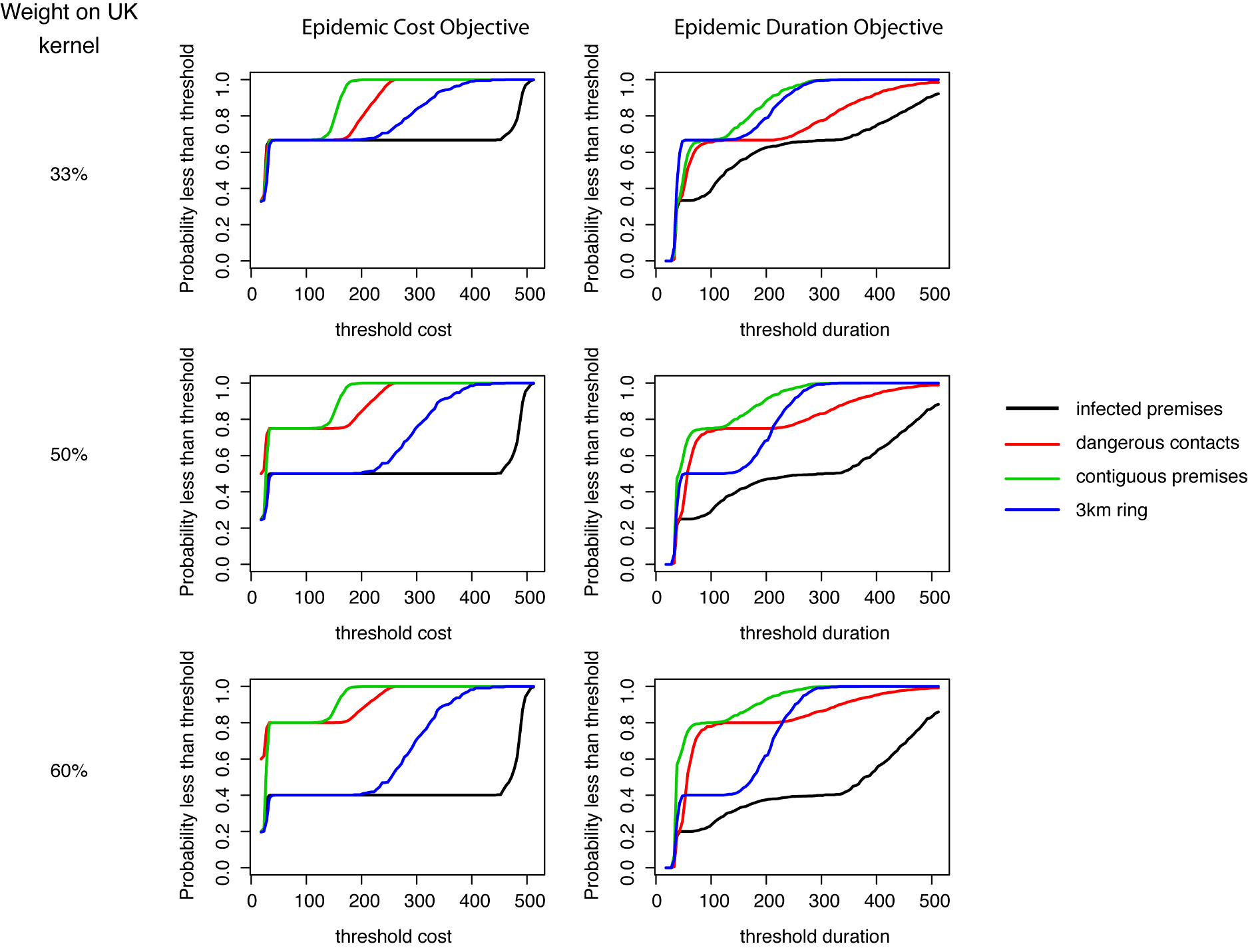

Supplement: Figure S1 — The probability of epidemic outcomes below a stated threshold for four alternative management tactics. Left panels give outcomes for the management objective to minimize total epidemic cost due to livestock loss, the right panels give outcomes for the management objective to minimize the duration of management activities. The x-axis indicates the cost (in millions of £) or duration (days) threshold that managers would like to stay below. The y-axis indicates the probability, averaged across all three kernel models, of outcomes below the threshold for each management tactic (solid lines). Panels from top to bottom indicate increasing weight on the 2001 UK kernel, with equal remaining weight on kernels 1 and 3. (TIF) [file pbio.1001970.s001.tif]

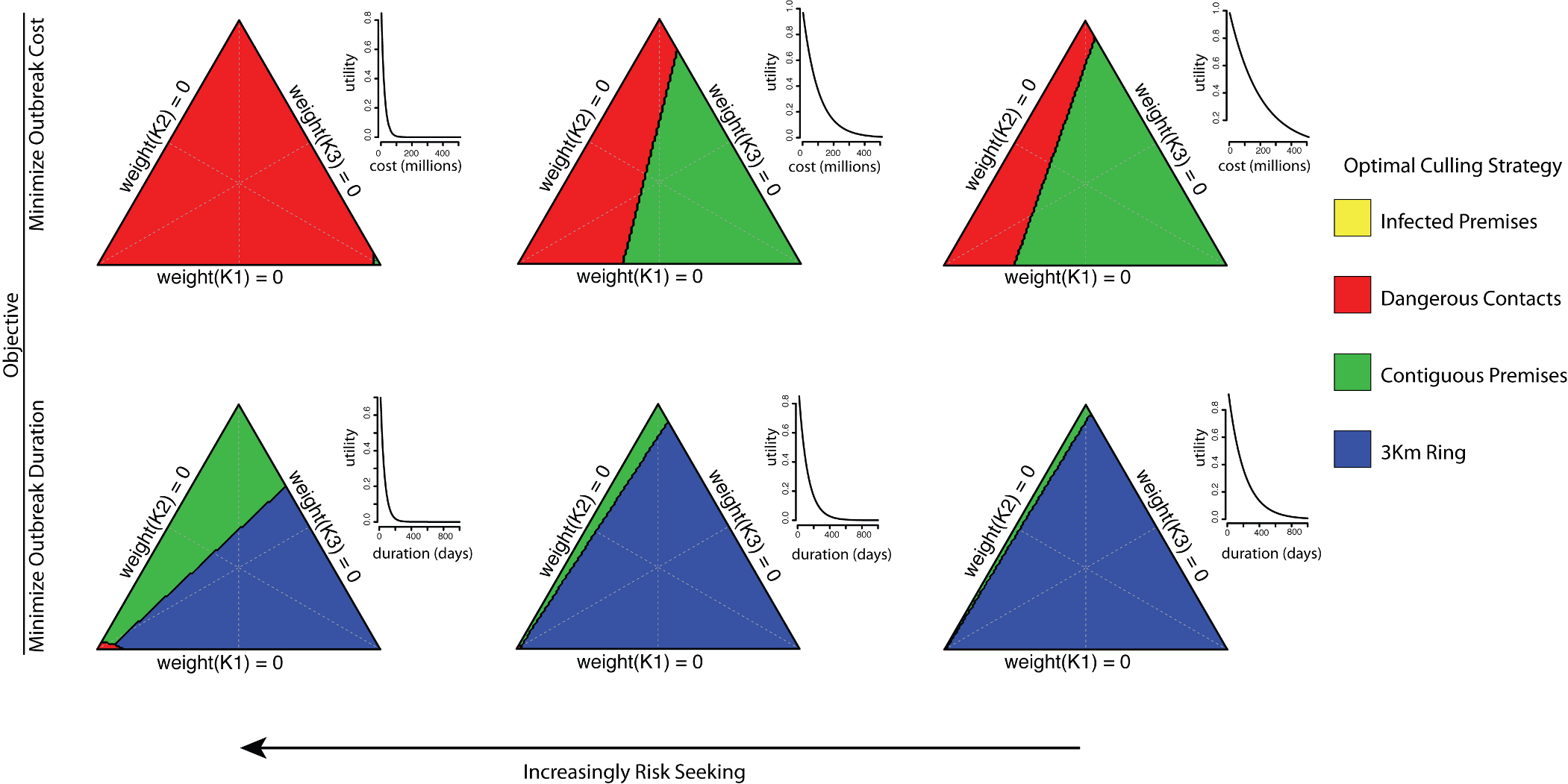

Supplement: Figure S2 — Ternary plots of the optimal static strategies assuming different utility functions. The top row indicates the optimal static strategy for the objective of minimizing total outbreak cost due to livestock loss. The bottom row indicates the optimal static strategy for the objective of minimizing outbreak duration. Each ternary figure indicates the optimal static culling alternative (colors) for different weightings on the three kernel models (see Figure 3 in the main text for description of ternary plots). Panels from right to left indicate utility functions (insets) that are increasingly risk-seeking. (TIF) [file pbio.1001970.s002.tif]

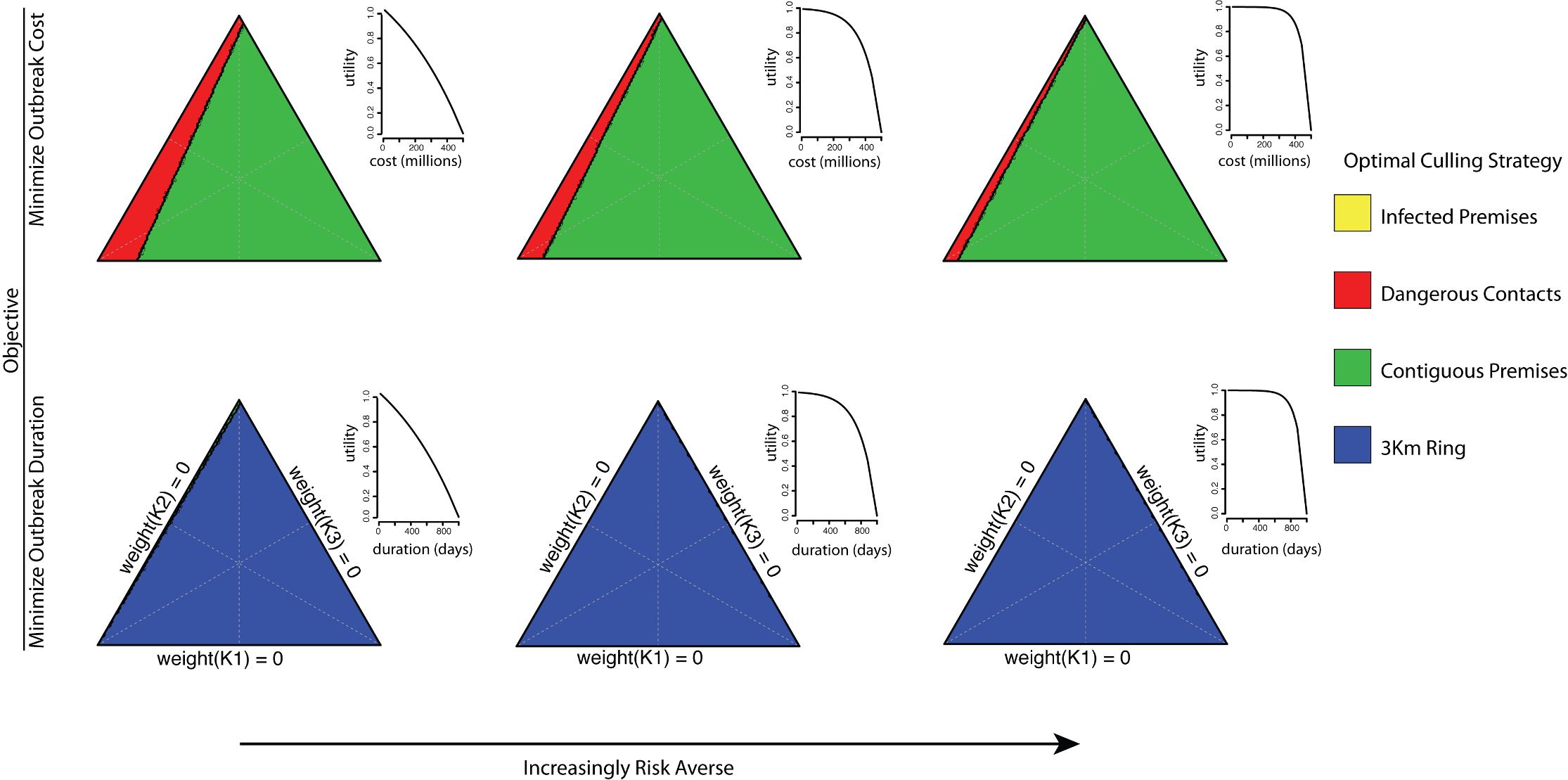

Supplement: Figure S3 — Ternary plots of the optimal static strategies assuming different utility functions. The top row indicates the optimal static strategy for the objective of minimizing total outbreak cost due to livestock loss. The bottom row indicates the optimal static strategy for the objective of minimizing outbreak duration. Each ternary figure indicates the optimal static culling alternative (colors) for different weightings on the three kernel models (see Figure 3 in the main text for description of ternary plots). Panels from left to right indicate utility functions (insets) that are increasingly risk-averse. (TIF) [file pbio.1001970.s003.tif]
