# Supplementary material for: Adaptive Management and the Value of Information: Learning Via Intervention in Epidemiology
Source: PLoS Biol. 2014 Oct 21;12(10):e1001970. doi: 10.1371/journal.pbio.1001970 (PMC4204804; doi:10.1371/journal.pbio.1001970)
Supplement: Table S1 — Cost projections (in millions of £) for each two-stage intervention strategy for each kernel model. (DOCX) [file pbio.1001970.s004.docx]

**Table S1.** Cost projections (in millions of £) for each 2-stage intervention strategy for each kernel model.

| Stage 1 Intervention | IP | | | | DC | | | | CP | | | | 3R | | | |
| --- | --- | --- | --- | --- | --- | --- | --- | --- | --- | --- | --- | --- | --- | --- | --- | --- |
| Stage 2 Intervention | IP | DC | CP | 3R | IP | DC | CP | 3R | IP | DC | CP | 3R | IP | DC | CP | 3R |
| K1 (thin) | 84 | 74 | 87 | 149 | 59 | 55 | 99 | 239 | 81 | 81 | 82 | 205 | 130 | 129 | 165 | 260 |
| K2 (UK) | 5129 | 2238 | 1676 | 3395 | 4991 | 1901 | 1285 | 2779 | 4662 | 1823 | 1162 | 2074 | 4917 | 1875 | 1284 | 2538 |
| K3 (fat) | 284 | 283 | 306 | 326 | 223 | 221 | 379 | 287 | 379 | 376 | 378 | 438 | 310 | 310 | 446 | 382 |
| Case 1 | 2657 | 1208 | 936 | 1816 | 2566 | 1020 | 762 | 1521 | 2446 | 1026 | **696** | 1198 | 2568 | 1047 | 795 | 1429 |
| Case 2 | 1833 | 865 | 690 | 1290 | 1758 | 726 | 588 | 1102 | 1708 | 760 | **541** | 906 | 1785 | 772 | 632 | 1060 |
