# Supplementary material for: Adaptive Management and the Value of Information: Learning Via Intervention in Epidemiology
Source: PLoS Biol. 2014 Oct 21;12(10):e1001970. doi: 10.1371/journal.pbio.1001970 (PMC4204804; doi:10.1371/journal.pbio.1001970)
Supplement: Table S2 — Expected cost projections (in millions of £) of each first-stage intervention, conditional on the assumption that model uncertainty is resolved after 1 month and the second-stage action is taken as that intervention that minimizes costs under the true model. (DOCX) [file pbio.1001970.s005.docx]

**Table S2.** Expected cost projections (in millions of £) of each first-stage intervention, conditional on the assumption that model uncertainty is resolved after 1 month and the second-stage action is taken as that intervention that minimizes costs under the true model.

|  |  | Management Actions in Stage 1 | | | |  |
| --- | --- | --- | --- | --- | --- | --- |
| Models | Kernel | IP | DC | CP | RC | Best |
|  | K1 (thin) | 73.5 | 55.1 | 80.5 | 129.3 | 55.1 |
|  | K2 (UK) | 1676.1 | 1285.5 | 1162.2 | 1284.4 | 1162.2 |
|  | K3 (fat) | 283.0 | 221.1 | 376.1 | 310.0 | 221.1 |
| Case 1 | UK kernel belief  (0.25, 0.50, 0.25) | *927.1* | 711.8 | **695.3** | 752.0 | 650.1 |
| Case 2 | Equal weighting  (0.33, 0.33, 0.33) | *677.5* | **520.5** | 539.6 | 574.6 | 479.5 |
