# Supplementary material for: Adaptive Management and the Value of Information: Learning Via Intervention in Epidemiology
Source: PLoS Biol. 2014 Oct 21;12(10):e1001970. doi: 10.1371/journal.pbio.1001970 (PMC4204804; doi:10.1371/journal.pbio.1001970)
Supplement: Table S3 — Parameterization of the age distribution of susceptibles for three age distribution models. (DOCX) [file pbio.1001970.s006.docx]

Table S3. Parameterization of the age distribution of susceptibles for three age distribution models.

| Model | Delta | Theta |
| --- | --- | --- |
| 90% susceptibles < 5 | 0.50 | 0.5 |
| 90% susceptibles <10 | 0.16 | 0.2 |
| 90% susceptibles <15 | 0.09 | 0.1 |
