# Supplementary material for: Adaptive Management and the Value of Information: Learning Via Intervention in Epidemiology
Source: PLoS Biol. 2014 Oct 21;12(10):e1001970. doi: 10.1371/journal.pbio.1001970 (PMC4204804; doi:10.1371/journal.pbio.1001970)
Supplement: Text S1 — Additional methods and model descriptions. (A) Description of the Warwick FMD model. (B) Interpretation of the EVPI table. (C) Expected value of an adaptive strategy. (D) Description of the measles outbreak model. (E) Alternate objective formulations to reflect risk tolerance for FMD. (DOCX) [file pbio.1001970.s007.docx]

# Supplementary Information Text

# A: The Warwick FMD model

In the Keeling *et al.* [[1](#_ENREF_1)] model, once a farm *i* becomes infectious, it infects farm *j* at a rate given by:

$$\text{Rate}_{ij}=\left(\left[N_{sheep,j}\right]^{p_{t}}S_{sheep}+\left[N_{cow,j}\right]^{p_{c}}S_{cow}\right)*\left(\left[N_{sheep,i}\right]^{q_{c}}T_{sheep}+\left[N_{cow,i}\right]^{q_{c}}T_{cow}\right)*\left(\mathrm{K}\left(d_{ij}\right)\right)$$

Here, *Ns,i* is the number of livestock species s recorded as being on farm i, Ss and Ts measure the species-specific susceptibility and transmissibility, *dij* represents the distance between infectious farm *i* and susceptible farm *j* and K is the distance-dependent transmission kernel. Here we consider the kernel estimated from contact tracing data from the 2001 epidemic after the introduction of movement restrictions (K2; [[1](#_ENREF_1)]) as well as 2 alternative kernels. Kernel K1 puts more weight on local transmission and was calculated as having 4-times the height of K2 and ¼ the width of K2. Kernel K3 puts more weight on long-distance transmission and was calculated as having ¼ the height of K2 and 4-times the width of K2.

As an extension to the model developed during 2001, power law parameters are introduced to allow for non-linear scaling in susceptibility and transmissibility as animal numbers on each farm increase. Previous work has shown that a model in which power laws are included provides a more accurate fit to the 2001 data than one in which powers are set to 1 [[2-4](#_ENREF_2)].

During the 2001 UK FMD epidemic, all farms with livestock confirmed to be infected with disease were designated infected premises, or IPs. Animals on these farms were routinely culled and in addition all dangerous contact farms (DCs) were pre-emptively culled as part of the disease control policy. DCs were defined either as premises in which animals had been in direct contact with infected livestock or as premises that had been exposed to infection in any other way. DCs in the model are determined based upon infection by an IP and upon likely risk of future infection in the same way as prior work [[5](#_ENREF_5)]. In the early stages of the UK FMD outbreak, IPs and DCs were often culled many days after reporting infection. By late March, additional measures were introduced in an effort to control the disease. It was decided that all IPs should be culled within 24 hours of reporting infection and all DCs within 48 hours. Additional localised culling was also performed, whereby all livestock on any farm sharing a border with an IP were culled. This culling policy became known as the Contiguous Premises (CP) cull. In this model, CPs are determined by tessellating around each farmhouse location, taking the area of each farm into account. A surrogate set of adjacent farms are thus obtained. In this paper all IPs are culled within 24 hours of reporting infection and all associated pre-emptive culls (DCs and CPs) within 48 hours.

The aim of this work is to determine the effectiveness of alternate policies for disease control based upon epidemic characteristics. In addition to culling of IPs, DCs and CPs, we therefore consider the effectiveness of a policy of 3km ring culling. Therefore, when an IP is reported all farms within 3km of that IP will be targeted for culling. Ring culling would be carried out in addition to IP and DC culling and we assume that the resources available to carry out any control policy are limited. A maximum of 100 farms can be ring culled per day in line with previous work. Sensitivity to these limits has been investigated in detail previously [[6](#_ENREF_6)].

**B: Interpretation of EVPI Table (Table 2)**

Here we provide a more thorough description of the simulation results and calculation of EVPI from Table 2.

Under kernel K1, the best action is DC culling, as the narrow dispersal kernel contains the epidemic in a small geographic region. DC is also the best action if you know the kernel model is K3; geographic spread is significant, but the small height of the kernel results in relatively few cases in high density regions. However, if you know the kernel model is K2 (UK), the best action is to cull infected premises, dangerous contacts and contiguous premises (CP). K2’s combination of significant local *and* long distance spread results in higher costs regardless of the chosen intervention. Given the proposed *a priori* weights on the models, the CP strategy gives the lowest model-weighted projected cost (£696.0M for a stronger belief in the 2001 UK kernel, K2, and of £540.7M for equal model weightings). Thus, while the DC strategy is better under 2 of the 3 models, the higher costs associated with K2 mean that the CP strategy would result in the lowest expected cost in light of model uncertainty.

Despite IP culling being the least expensive strategy to implement of the 4 considered here, it is insufficient to curtail FMD spread, even with the narrowest dispersal model assumed, so overall incurs higher costs than DC under all models (hence, IP is *dominated by* DC). RC is also never the best solution, is the worst strategy for K1 and K3, and is dominated by CP.

If we could resolve uncertainty prior to committing to an action, we would choose the best action under the true model. Prior to resolving uncertainty, we can calculate the expected value of taking the best action, using the *a priori* model weights; the model-weighted expected costs of the best strategy are £65.0.1M and £479.5M for Case 1 and 2, respectively.

For Case 1 (UK kernel bias), the difference between the cost of the best single strategy in the face of uncertainty, given the model weights (CP: £696.0M) and the model-weighted average of the best strategy, conditional on each model (£650.1M) is £45.9M. Thus, the expected cost of a future outbreak could be reduced by £45.9M (6.6%) if the uncertainty could be fully resolved before a management action is decided.

For Case 2 (equal weighting), EVPI is £61.2M (=540.7M-47.9.5M), which is an 11.3% cost reduction. In practice, discrimination between these alternative kernels is unlikely to be possible prior to the observation of a novel outbreak. Thus, the EVPI quantifies the economic incentive for implementing an adaptive management plan.

**C: Expected Value of Adaptive Strategy**

We can calculate the expected value of an adaptive strategy, EVadaptive, as the reduction in costs associated with taking the two-stage strategy over the optimal strategy in the face of uncertainty. Here we define

$EV_{adaptive}=\min_{ij} \sum_{k} p_{k}C_{ijk}-\min_{i} \sum_{k} p_{k}C^{*}_{ik}$ Eq 1

$$C_{ik}^{*}=\min_{j} C_{ijk}$$

where Cijk is the projected cost of tactic *i* in the first stage and tactic *j* in the second stage under model *k*, and C*ik is the minimum cost over all second stage actions (*j*) when action *i* is the first stage action under model *k*.

Table S1 presents the projected costs Cij­ for each 2-stage intervention combination and each kernel model. The lowest cost combination (left hand term in Eq 1), averaged over model weights, is highlighted in bold in the bottom rows for each of the two example weightings. Table S2 presents the expected costs of each stage 1 intervention conditional on the assumption that model uncertainty is resolved after 1 month and the second-stage action is taken as that intervention that minimizes costs under the true model. The stage 1 intervention with the lowest expected cost conditional on model weights (right hand term in Eq 1) is highlighted in bold for each of the two example weightings. The EV­adaptive is calculated as the difference between the bold value in Table S2 and the bold value in Table S1.

**D: Measles outbreak model**

To explore age-targeting decisions for a measles outbreak, we developed a deterministic age-structured Susceptible-Exposed-Infected-Removed (SEIR) model. Infection was assumed to result in a 7-day exposed period in which individuals were not infectious, followed by a 7-day infectious period. The population was assumed to follow the age distribution from the 2008 national census in Malawi with individuals distributed in the following age classes: 0-6 mo, 6-12 mo, 1-2y, 2-3y, . . , 19-20y, and >20y. The force of infection was assumed to be constant in all age classes and mixing was assumed to be mildly assortative such that individuals have a higher probability of infecting individuals of the same age; to achieve the latter, we assumed a Who Acquires Infection From Whom (WAIFW) of the form

.

We assumed that all individuals < 6m of age had maternal immunity. The proportion of each of the remaining age classes that were susceptible to measles was assumed to decay exponentially; i.e.

$P(\text{age class a susceptible)}=\delta\exp(-\theta a)$.


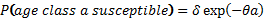

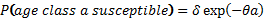


The transmission scaling term β was chosen to give an epidemic peak at 165 days in the absence of vaccination, which is consistent with the time course of the 2010 epidemic in Malawi (the epidemic peak in districts with no vaccination campaigns was on week 24 of the year). For the purposes of simplicity, birth and death were ignored in the model. Thus, the epidemic can be described by the following set of ordinary differential equations:

$$\begin{matrix} \frac{dS_{i}}{dt}=-\beta_{t}\sum_{j=1}^{n} A_{ij}S_{i}I_{j}-\alpha_{t}S_{i} \\ \frac{dE_{i}}{dt}=\beta_{t}\sum_{j=1}^{n} A_{ij}S_{i}I_{j}-\gamma E_{i} \\ \frac{dE_{i}}{dt}=\gamma E_{i}-\theta I_{i} \\ \frac{dR_{i}}{dt}=\theta I_{i}+\alpha_{t}S_{i} \end{matrix}$$

where S, E, I, and R are susceptible, exposed, infected, and recovered individuals respectively and the index, *i*, indicates the age class. Aij­ indicates the transmission from age class *j* to age class *i* (i.e. element *i, j* in the WAIFW matrix). The vaccination rate, $\alpha_{t}$, is the daily vaccination rate for age classes within the age target and is 0 if *t*<75 days or 95% of the susceptibles in the age target had been vaccinated. The parameters and $\theta$are the rate at which individuals leave the exposed and infectious classes, respectively; both were set to 1/7 to give a mean infectious generation of 14 days.


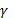

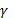


We considered 3 alternative models of the initial age distribution of susceptible individuals in the population of the form $\Pr(\text{susceptibles in age a})=\delta\exp(-\theta a)$ that gave 90% of the susceptible population less than 5, 10, or 15 years of age respectively (see Table S3 for parameters). See Figure 5A in the main text for the initial age distribution of susceptibles under each model.

Vaccination was assumed to begin in day 75. Individuals within the target age range were vaccinated at a set daily rate until the number of vaccine doses administered equaled 95% of the population size in the target age range. Thus, the duration of the vaccination campaign scales with both the daily vaccination rate and the target age range. We considered vaccination campaigns that targeted 95% of susceptibles less than 5, 10, or 15 years.

We calculated the EVPI and the best static age target for vaccination (as in Table 1, Eq 1 in the main text) for daily vaccination rates ranging from 10000/day to 100000/day assuming equal initial weights on the three age distribution models.

**E: Alternate Objective Formulations to Reflect Risk Tolerance**

In the main text, we assumed that the objective of FMD outbreak management was to minimize the expected total cost (where the expectation was taken over all the forms of uncertainty). This objective does not explicitly account for variation in outbreak outcomes. In particular, for epidemic settings, the distribution of potential outcomes may be strongly skewed, such that the majority of outcomes are of moderate cost but very extreme outcomes (e.g. a global pandemic) are possible. The risk tolerance of society to these extreme, but low probability, outcomes may be an important element of the management decision; thus, alternative management objectives may seek to minimize the likelihood of these highly adverse outcomes. The structured decision making framework presented in the main text can accommodate such objectives and we present an analysis of the FMD outbreak with respect to two alternate formulations of the objectives here.

Rather than seeking to minimize the expected (average) outcome, one may wish to specify the goals of management in terms of maximizing the probability of a “good” outcome (or conversely minimizing the probability of an adverse outcome), where “good” is defined as a quantitative threshold. Then, for any given threshold, we would choose the alternative that maximizes the likelihood of achieving an outcome below that threshold, T. If there were no model uncertainty, this would be a straightforward choice of the management tactic, *j*, that maximizes the probability that the outcome is less that T. However, if there are competing models, M, we can restate this decision criterion as:

$\max_{j} \sum_{m} \left( P(x<T|M=m,J=j)P(M=m) \right)$.

Thus, the choice of management tactic will depend on both the weights associated with each model, P(M=m), and the distribution of outcomes under each model and tactic.

The results for the FMD example are presented in Figure S1 for threshold objectives based on total cost or epidemic duration. We present results for the cases where the model weight on the UK dispersal kernel is either 33%, 50%, or 60% with equal weight on kernels 1 and 3. For both objectives (total cost and epidemic duration), the Contiguous Premises culling alternative either maximizes, or results in the same, likelihood of remaining below the threshold cost or duration regardless of kernel weighting for the majority of possible thresholds. However, when the threshold cost is very low and the weight on the UK kernel is high, the Dangerous Contacts culling alternative is more likely to remain below the threshold; this makes intuitive sense as the CP culling option is necessarily more aggressive and will result in greater losses due to preemptive culling. Similarly, if the weight on the UK kernel is low and the threshold duration is low, then the 3 km ring culling alternative is most likely to stay below the threshold; again this is intuitive as this is the more aggressive alternative. It is worth noting, however, that while these alternatives might be the most likely to result in outcomes below the threshold, that actual probability of exceeding the threshold may still be quite high.

Of course, the choice of threshold is itself a critical management decision. Rather than choosing a fixed threshold, one can define a utility function that transforms the outcomes from the scale of the objective function to a continuous measure of utility. The decision-maker then seeks to choose the management alternative that maximizes utility. A utility function that is concave on the scale of the objective guards against very bad outcomes by weighting them quite heavily, and is fairly indifferent to the differences in good outcomes. In this sense, such a utility function would reflect risk-averse decision making; with a premium put on avoiding very undesirable outcomes (e.g. high costs or long duration of an outbreak). A utility function that is convex on the scale of the objective puts a premium on the best outcomes by weighting them heavily, and remains indifferent to the differences in bad outcomes. Such a utility function would reflect a setting in which the decision-maker wants to minimize the objective but is willing to accept a range of outcomes except for the most costly; in this sense this would reflect risk-seeking decision making. Unlike a threshold, the utility function approach does not make a binary distinction between similar outcomes that lie on either side of a fixed point. However, the statement of the utility function itself requires a very clear statement of the goals and risk tolerance of the decision-maker.

Here we show, for the FMD example, the impact of increasingly risk-averse decision making via a utility function. For heuristic purposes, we chose a simple exponential utility function that states the utility, U, in terms of the objective, X:

$$U=e^{-\theta X}$$

where *θ* controls the concavity of the utility function; as *θ* increases, decision-making becomes increasingly risk-seeking. Figure S2 shows the optimal static (i.e. non adaptive) strategy to maximize utility for three levels of risk aversion for an objective to minimize outbreak cost due to livestock loss and an objective to minimize outbreak duration. When the decision-maker is relatively risk neutral (i.e. the utility function is nearly linear in terms of the objective), the optimal decision is the same as the optimal static decision in the absence of the utility transformation. As a decision-maker with the objective of minimizing costs becomes increasingly risk-seeking, the Dangerous Contacts culling alternative becomes optimal for a broader range of kernel weightings. As a decision-maker with the objective of minimizing outbreak duration becomes increasingly risk-seeking (i.e. right to left in Figure S2), the Contiguous Premises culling alternative becomes optimal when the weight on the UK kernel (K2) is low. Note that while the CP culling alternative does maximize utility for some weightings, the difference in utility (with respect to epidemic duration) between the CP and 3 km ring culling option is minor. Figure S3 shows the same, for a setting in which the decision-maker is risk-averse. As the utility function becomes increasingly concave (i.e. more severely deprecating the utility of extreme outcomes relative to all others – left to right in Figure S3) the decision space becomes increasing dominated by the most conservative alternative; CP culling if the objective is to minimize costs, and ring culling if the objective is to minimize outbreak duration.

References

1. Keeling MJ, Woolhouse MEJ, Shaw DJ, Matthews L, Chase-Topping M, et al. (2001) Dynamics of the 2001 UK foot and mouth epidemic: Stochastic dispersal in a heterogeneous landscape. Science 294: 813-817.

2. Diggle PJ (2006) Spatio-temporal point processes, partial likelihood, foot and mouth disease. Statistical Methods in Medical Research 15: 325-336.

3. Tildesley MJ, Deardon R, Savill NJ, Bessell PR, Brooks SP, et al. (2008) Accuracy of models for the 2001 foot-and-mouth epidemic. Proceedings of the Royal Society B-Biological Sciences 275: 1459-1468.

4. Deardon R, Brooks SP, Grenfell BT, Keeling MJ, Tildesley MJ, et al. (2010) Inference for individual-level models of infectious diseases in large populations. Statistica Sinica 20: 239-261.

5. Tildesley MJ, Savill NJ, Shaw DJ, Deardon R, Brooks SP, et al. (2006) Optimal reactive vaccination strategies for a foot-and-mouth outbreak in the UK. Nature 440: 83-86.

6. Tildesley MJ, Keeling MJ (2008) Modelling foot-and-mouth disease: A comparison between the UK and Denmark. Preventive Veterinary Medicine 85: 107-124.
